# Supplementary material for: KDM5D Histone Demethylase Identifies Platinum-Tolerant Head and Neck Cancer Cells Vulnerable to Mitotic Catastrophe
Source: Int J Mol Sci. 2023 Mar 10;24(6):5310. doi: 10.3390/ijms24065310 (PMC10049674; doi:10.3390/ijms24065310)
Supplement: Supplementary file 1 [file ijms-24-05310-s001.zip › ijms-2192386-supplementary.pdf]

SUPPLEMENTARY INFORMATION

KDM5D Histone Demetyhlase Identifies Platinum-Tolerant Head and Neck Cancer Cells Vulnerable to Mitotic Catastrophe

Tsung-Ming Chen[1,2,] Chih-Ming Huang[3,4,] Syahru Agung Setiawan[5,6,] Ming-Shou Hsieh[7,8,9,] Chih-Chi Sheen[7,8,9*,] Chi-Tai Yeh[6,10*]

[1] Department of Otolaryngology, School of Medicine, College of Medicine, Taipei Medical University, Taipei City 11031, Taiwan

[2] Department of Otolaryngology-Head and Neck Surgery, Shuang Ho Hospital, Taipei Medical University, New Taipei City 23561, Taiwan

[3] Department of Otolaryngology, Taitung Mackay Memorial Hospital, Taitung City 950408, Taiwan

[4] Department of Nursing, Tajen University, Yanpu 90741, Pingtung County, Taiwan

[5] International Ph.D. Program in Medicine, College of Medicine, Taipei Medical University, Taipei City 11031, Taiwan

[6] Department of Medical Research & Education, Taipei Medical University-Shuang Ho Hospital, New Taipei City 23561, Taiwan

[7] School of Dentistry, College of Oral Medicine, Taipei Medical University, Taipei City, 110, Taiwan.

[8] Department of Dentistry, Taipei Medical University-Shuang Ho Hospital, New Taipei City, 235, Taiwan.

[9] Department of Periodontics, Shuang Ho Hospital, Taipei Medical University, New Taipei City 23561, Taiwan.

[10] Continuing Education Program of Food Biotechnology Applications, College of Science and Engineering, National Taitung University, Taitung 95092, Taiwan.

*Authors to whom correspondence should be addressed.

Chi-Tai Yeh, PhD

Department of Medical Research and Education, Taipei Medical University - Shuang Ho Hospital, New Taipei City 23561, Taiwan. Tel: +886-2-2490088 ext. 8881, Fax: +886-2-2248-0900. E-mail: ctyeh@s.tmu.edu.tw

Chih-Chi Sheen, MD

Department of Periodontics, Shuang Ho Hospital, Taipei Medical University, New Taipei City 23561, Taiwan. Tel: +886-2-2490088 ext. 8885, Fax: +886-2-2248-0900. E-mail:15484@s.tmu.edu.tw

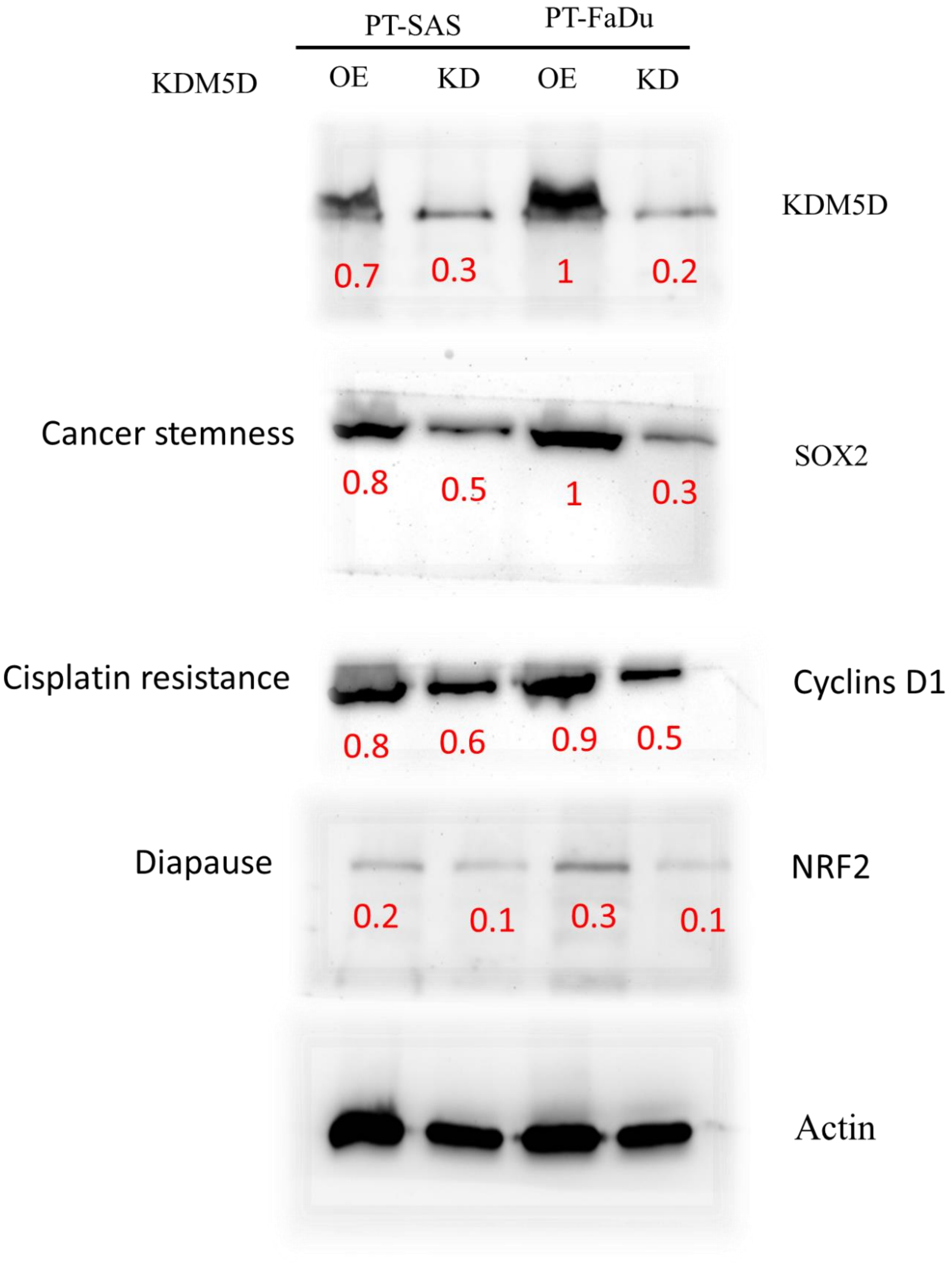

Supplementary Figure S1 KDMD5 and AURKB overexpression/knockdown with an established positive marker for cancer stemness (SOX2), cisplatin resistance (Cyclins D1) and diapause (NRF2)

.

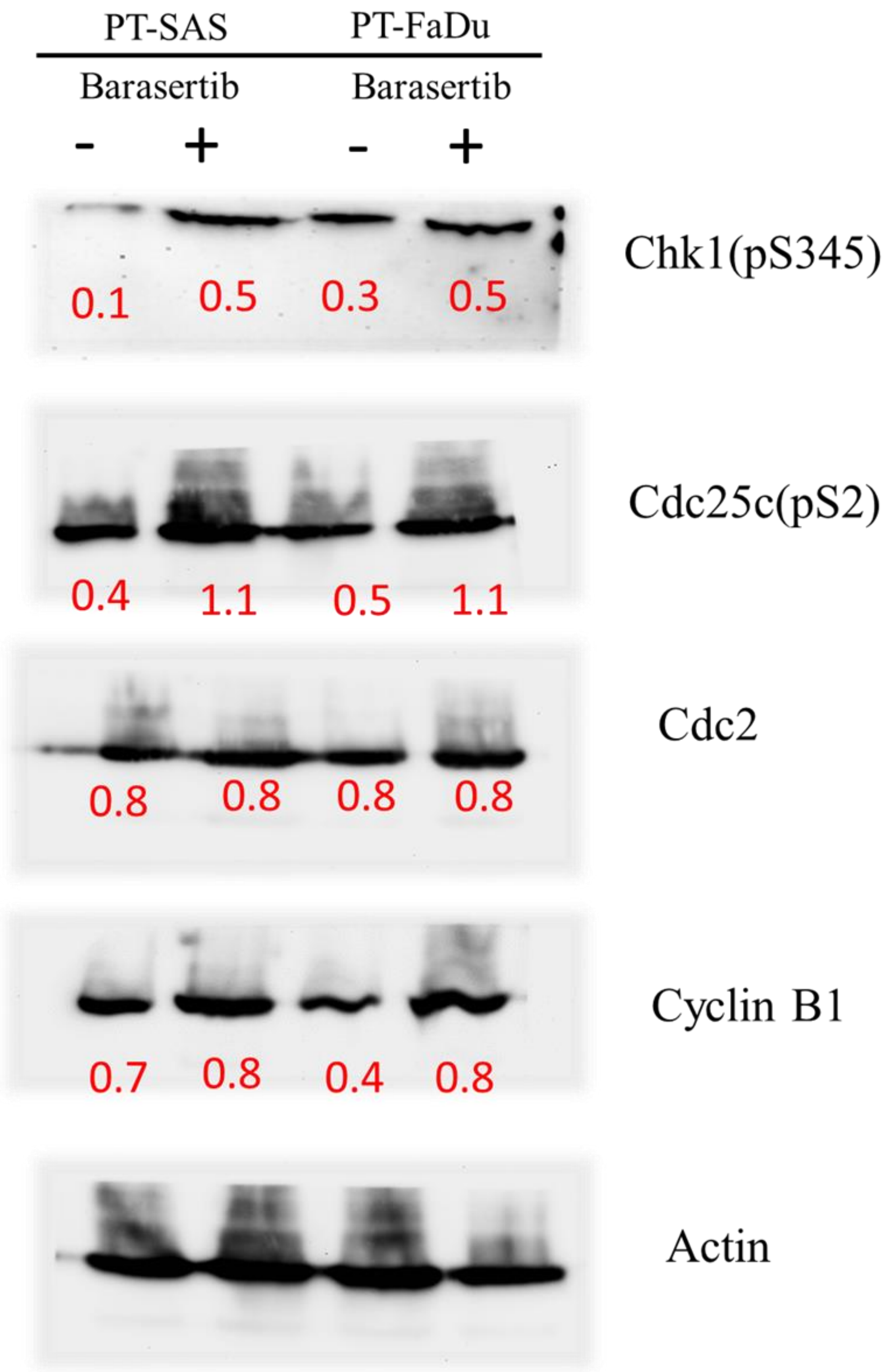

Supplementary Figure S2. Full-size blots of Figure 6D
